# Assessing the likelihood of contracting COVID-19 disease based on a predictive tree model: A retrospective cohort study

**DOI:** 10.1371/journal.pone.0247995

**Published:** 2021-03-03

**Authors:** Francesc X. Marin-Gomez, Mireia Fàbregas-Escurriola, Francesc López Seguí, Eduardo Hermosilla Pérez, Mència Benítez Camps, Jacobo Mendioroz Peña, Anna Ruiz Comellas, Josep Vidal-Alaball

**Affiliations:** 1 Health Promotion in Rural Areas Research Group, Gerència Territorial de la Catalunya Central, Institut Català de la Salut, Barcelona, Spain; 2 Unitat de Suport a la Recerca de la Catalunya Central, Fundació Institut Universitari per a la Recerca a l’Atenció Primària de Salut Jordi Gol i Gurina, Barcelona, Spain; 3 Sistemes d’Informació dels Serveis d’Atenció Primària, Institut Català de la Salut, Barcelona, Spain; 4 Departament de Ciències Experimentals, Grup d’Investigació Economía i Salut, Pompeu Fabra University, Barcelona, Spain; 5 Sistema de Informació pel Desenvolupament d’Investigació en Atenció Primària, Institut Universitari d’Investigació en Atenció Primària Jordi Gol, Barcelona, Spain; 6 Equip d’atenció Primària Gòtic, Institut Català de la Salut, Barcelona, Spain; 7 Departament de Salut, Direcció i Coordinació de la Resposta a la COVID19, Generalitat de Catalunya, Barcelona, Spain; 8 Equip d’atenció Primaria Sant Joan de Vilatorrada, Institut Català de la Salut, Barcelona, Spain; Universidad Nacional de la Plata, ARGENTINA

## Abstract

**Background:**

Primary care is the major point of access in most health systems in developed countries and therefore for the detection of coronavirus disease 2019 (COVID-19) cases. The quality of its IT systems, together with access to the results of mass screening with Polymerase chain reaction (PCR) tests, makes it possible to analyse the impact of various concurrent factors on the likelihood of contracting the disease.

**Methods and findings:**

Through data mining techniques with the sociodemographic and clinical variables recorded in patient’s medical histories, a decision tree-based logistic regression model has been proposed which analyses the significance of demographic and clinical variables in the probability of having a positive PCR in a sample of 7,314 individuals treated in the Primary Care service of the public health system of Catalonia. The statistical approach to decision tree modelling allows 66.2% of diagnoses of infection by COVID-19 to be classified with a sensitivity of 64.3% and a specificity of 62.5%, with prior contact with a positive case being the primary predictor variable.

**Conclusions:**

The use of a classification tree model may be useful in screening for COVID-19 infection. Contact detection is the most reliable variable for detecting Severe acute respiratory syndrome coronavirus 2 (SARS-CoV-2) cases. The model would support that, beyond a symptomatic diagnosis, the best way to detect cases would be to engage in contact tracing.

## Introduction

On 31 December 2019, the authorities of the People’s Republic of China reported numerous cases of pneumonia of unknown etiology in Wuhan (China) to the WHO. A week later they confirmed that it was a new coronavirus outbreak that has been called SARS-CoV-2 [1, 2]. The outbreak then spread beyond China’s borders, affecting most countries in the world until the WHO declared the outbreak a pandemic on 12 March 2020 [3]. Most of the cases (approximately 80%) of COVID-19 reported so far are mild and, at present, there is no specific treatment, meaning the clinical approach is symptomatic treatment in mild to moderate cases and support measures or treatment of complications in severe cases [4].

In Catalonia, the spread of the pandemic has put a severe strain on the health system, which has had to adapt. This impact has been especially important in primary care centres, which are the main point of access for COVID-19 cases (health centres, continuing care facilities, primary care emergency centres, rural health clinics and so on). One of the strengths of the primary care system in Catalonia is the existence of a computerized medical history which covers 95% of a population of approximately seven and a half million people [5]. This has made it possible to access systematic and reliable information on the health status of the public throughout the pandemic and the results of PCR tests carried out during this time. These IT systems are complemented by diagnostic support tools. The high transmissibility of COVID-19 has meant that extreme precautionary measures must be taken, and diagnostic support tools must be used while exposing healthcare professionals to the lowest possible risk [4].

Clinical decision support systems (CDSS) play an important role in medicine, especially in medical diagnostic processes, and these are becoming increasingly important in medical decision making, particularly in those situations in which decisions must be made effectively and reliably [6]. Binary decision trees are good examples of CDSS, since they can be used by conceptual and machine learning-based decision-making models which have been demonstrated to be good tools to perform such tasks. They are being used successfully for many clinical purposes and could also be very useful in addressing suspected cases of COVID-19 infection: various trials have already been conducted at the hospital level, such as the use of a normogram to predict the risk of serious disease in the screening of new patients [7] or in primary care, to predict the prognosis of the disease [8]. Algorithms have also been proposed that include the use of computed tomography scans and laboratory testing [9, 10] and even mobile apps are being developed for smartphones to help assess the risk of infection [11].

In this context, the aim of this study is to evaluate the application of a decision tree to the diagnosis of COVID-19 based on clinical and sociodemographic information registered in the medical history collected by the primary care system in Catalonia.

## Methods

### Data collection sheet design

Primary care professionals have been using a COVID-specific data collection sheet since 26 March 2020, as part of the computerized medical history used by the primary care system, which is intended to identify patients who present with symptoms compatible with a coronavirus infection. This sheet was set up to conduct face-to-face or telephone triage of patients, electronically document related clinical information, manage and communicate the results of pandemic-related tests, and oversee the safe handling of such cases. It automatically accesses the demographic data in the patient’s medical history (sex, age and the existence of risk factors, among others) in order that the healthcare professionals treating the patient need only record the possible presence of contact with infected individuals and associated symptoms such as fever, cough, shortness of breath, a general feeling of being unwell, an altered mental state, gastrointestinal symptoms (including vomiting and diarrhoea), anosmia / ageusia, (i.e., loss of smell / loss of taste) and other symptoms and signs.

### Sample

**Fig 1:**
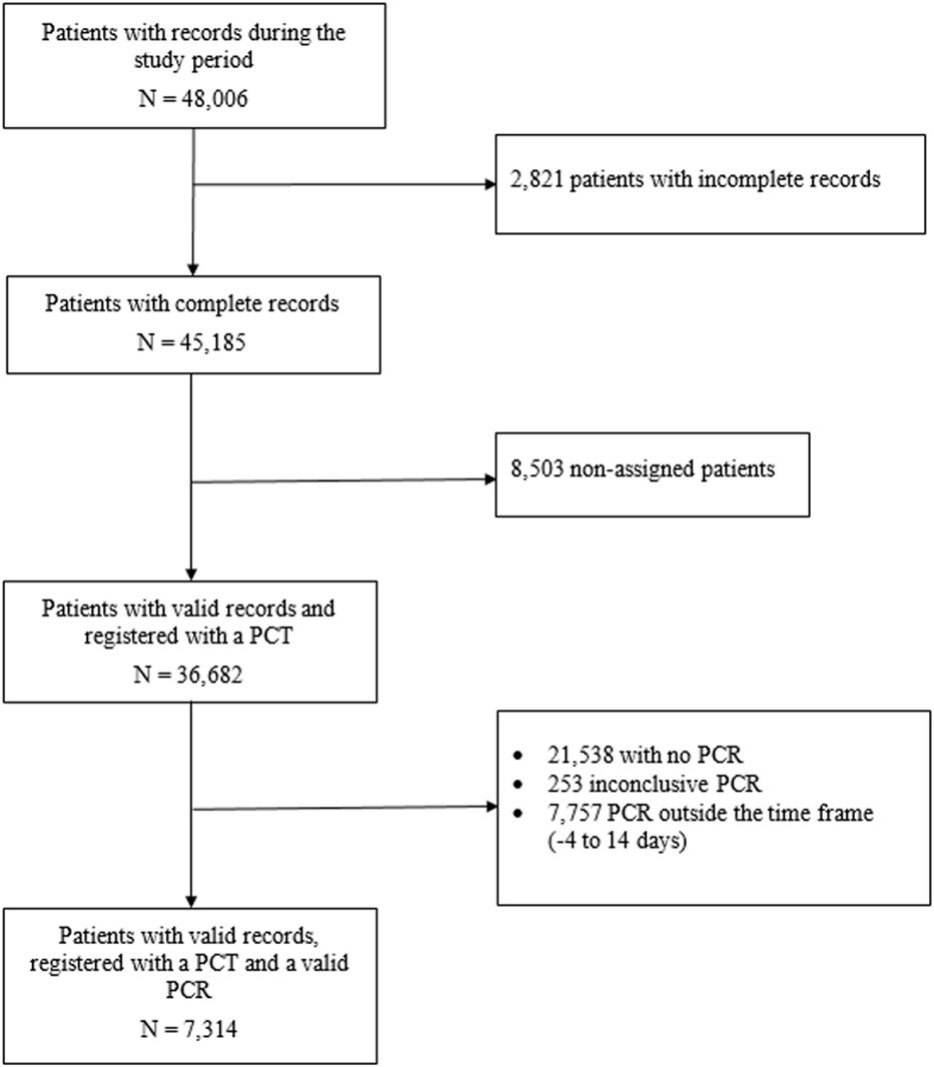
Participant flowchart.

This study includes a retrospective cohort of individuals assigned to one of the 311 Primary Care Teams (PCT) run by the Catalan Institute of Health (the provider of primary care services for three quarters of the population of Catalonia [5]), with an active medical history, for whom the COVID-19 screening sheet was completed and who had been subject to a SARS-CoV-2 PCR analysis, up to 14 days after registration. The study period ran from 26 March to 30 April 2020, the period with the highest incidence of SARS-CoV-2 in Catalonia. The only source of data is the anonymised records of patients who attended primary care teams. Data used was collected for the first time in a same participant with a PCR result close to the screening sheet record. During the study period, 67,128 valid assessments were performed on the record sheet, which corresponded to 36,682 different individuals. 15,144 of these (41.3%) underwent a SARS-CoV-2 RT-PCR, though the test was performed within 14 days of the assessment in only 7,314 (19.9%) cases (Fig 1).

### Study variables

The main variable of interest is the result (positive or negative) of the Reverse transcription-PCR test among the members of the cohort studied. From the samples collected with a nasal and throat swab with Virus Transport Medium (VTM) refrigerated at 4° C for a maximum of 48 hours, the Cobas © SARS-CoV-2 technique was performed, which has sensitivity and specificity close to 100% [12].

#### Descriptive statistics

For the descriptive analysis, the variables recorded at the time of the visit and those which are automatically collected from the patient’s medical history such as age, sex, the presence of comorbidities and other important underlying conditions such as rurality, socioeconomic level and morbidity clusters [13]. To assess the socioeconomic level, the deprivation index [14] was used, in which R refers to living in a rural area and U in an urban area. An urban area is considered to be a municipality with more than 10,000 inhabitants and a population density greater than 150 / km2. Urban areas are sub-classified by quintiles between U1 and U5, ranging from least (U1) to greatest (U5) deprivation. This index has been used to establish an association between socioeconomic indicators and mortality in different cities [15]. The following comorbidities were considered: cerebrovascular disease (including dementia and stroke), cancer (solid organ or haematological neoplasms diagnosed in the last 5 years), presence of immunosuppression, chronic renal failure, chronic respiratory disease (including chronic bronchitis / emphysema and asthma), heart disease (including congestive heart failure, coronary artery disease, atrial fibrillation, and other heart diseases), liver disease (including chronic hepatitis and cirrhosis), hypertension and diabetes mellitus. The question that arises in a multivariate context is the determination of the best explanatory model, which means evaluating the relevance of the different independent variables initially proposed in the regression model and choosing those that best explain the dependent variable. For this purpose, an analysis of the association between the different variables collected and the dependent variable PCR positive was performed.

#### Logistic model

A multinomial logit model was used for a full factorial model through the SPSS Statistics Standard Edition program. The estimation of the parameters was carried out through an iterative maximum likelihood algorithm. Although the ratio of the advantages of any pair of categories was assumed to be independent of the other response categories, an adjustment was made for the variables age and sex. To find out if there is a relationship between different categorical variables collected and the fact of presenting a positive PCR test (dependent variable), the independent variables that were shown to be related to the response variable in the descriptive analysis were used as possible predictors and that therefore could be good predictors. In particular, the variables that were studied as possible predictors were age, gender, rurality, socioeconomic level, the presence of chronic diseases and symptoms, according to type and number. The adequacy of the model was based on its ability to show an improvement with respect to the null model (model without predictors). From the 3 statistical tests that quantify this improvement (likelihood ratio, score and Wald test) we choose the likelihood ratio.

#### Decision tree

Prediction results were obtained using the QUEST classification type of tree. This method is fast and avoids other methods’ bias in favor of predictors with many categories as in the study. This kind of decision tree can be specified only if the dependent variable is nominal (as PCR result). The predictor variables proposed for the logistic model were the same as those used to make the decision tree model.

#### ROC curve

To check the degree of success of the two classification methods (regression model and tree model), we used the ROC curves functions from the R package pROC, for statistics. An estimate of the area under the curve was made using a binegative exponential model, and the ROC curve was obtained, with its confidence interval and coordinate points. The quantitative contrast variables were obtained from the probabilities resulting from the regression analysis and the tree model. To avoid introducing a bias in test using train-data, we performed a train-test split before data preparation steps. To simulate a train and test set we split randomly the data set into 80% train and 20% test.

### Ethical considerations

The study was approved by the Ethics Committee for Clinical Research (ECCR) of the Jordi Gol University Institute for Primary Care Research (IDIAP) in Barcelona (Code 20/088-PCV) and was carried out in accordance with the general ethical principles for observational studies [16]. The ethics committee has waived the requirement of informed consent given that most of the information related to Covid-19 in Catalonia that has been used in the study is open data that can be consulted from the Catalan government web [17, 18]. Contained data has gone through a process of anonymization and to avoid risks of re-identification some data have been generalized (age has been added in age groups) or eliminated to guarantee the confidentiality of sensitive data in technical applications of anonymization.

### Statistical analysis

The continuous variables are presented as mean and standard deviations. The categorical variables are presented as counts and percentages and the comparison between groups was done using the Chi-square independence test with the Yates continuity correction or the Fisher exact test, as appropriate. The logistic regression model is adjusted for assessing the association of the different values/variables with the probability of having a positive test of SARS-CoV-2, after adjusting for age, sex and other symptoms. The results of the regression are calculated as odds ratios (OR) and the corresponding confidence intervals (CI) of 95%. The Hosmer-Lemeshow test was also used with 10 groups as a fit-test of the model. The QUEST algorithm was used to grow the classification tree. The predictive accuracy of each model was evaluated by calculating the area under the curve (AUC).

The level of statistical significance was considered to be p <0.05 (bilateral). The IBM SPSS Statistics for Windows package (version 24) and the R package with RStudio (version 4.2.1) were used for the analysis.

## Results

### Demographic and clinical characteristics

The mean age of the participants in the study was 51.5 ± 19.1 years, with the participants who had a positive PCR (p <0.001) being significantly older. 42.79% (3,686/7,413) had positive PCR. The proportion of men with positive tests was significantly higher than women (52.0% vs. 49.5%) (p = 0.041). Regarding the social variables, although no association is observed between the socioeconomic level of those evaluated and the results of the PCR, the rurality variable did have an association, with a lower number of positives (24.1% vs. 27.8%) in rural areas (p <0.001). Those who tested positive for SARS-CoV-2 do not show a higher number of chronic diseases, but do show symptoms with a significantly higher frequency than those with negative tests: fever (33.5% vs. 23.6%), cough (37.7% vs. 34.5%), diarrhoea (19.0% vs. 17.0%), slurred speech (3.9% vs. 2.1%), a general feeling of being unwell (49.3% vs. 39.2%), an altered mental state (2.6% vs. 1.8%) and anosmia / ageusia (27.4% vs. 13.2%) (all p < 0.05). Most part of participants with a valid PCR (71.8%) had symptoms (including fever, cough, dyspnoea, pleurisy, hemoptysis, vomiting and diarrhoea) and 53.7% of them had a positive PCR (p < 0.001), a greater number of symptoms was associated with a positive PCR (an average of 2.43±1,35 compared with 2.28±1.26) (p < 0.001). 67.8% (2,345/3,953) of the subjects who reported having been in contact with a confirmed case of COVID-19 tested positive for SARS-CoV-2 (p < 0.001) (Table 1).

**Table 1:** Distribution of COVID-19 cases confirmed (positive PCR) or excluded (negative PCR), according to clinical and sociodemographic characteristics.

Characteristics n (%) or Mean (SD)		Valid PCR (N = 7,314)	Positive PCR (N = 3,686)	Negative PCR (N = 3,628)	p value
**Age [0–104]**		51.54 (19.1)	52.45 (18.6)	50.43 (19.6)	<0.001
**Sex**	**Male**	2,592 (35.4)	1,348 (36.6)	1,244 (34.3)	0.041
	**Female**	4,722 (64.6)	2,338 (63.4)	2,384 (65.7)	
**Rurality**		1,896 (25.9)	888 (24.1)	1,008 (27.8)	<0.001
**Socioeconomic level (0–82)**		43.5 (14.5)	43.87 (14.05)	43.13 (14.92)	0.079
**Chronic diseases**^b^	**Diabetes mellitus**	675 (9.2)	360 (9.8)	315 (8.7)	0.108
	**Hepatopathy**	24 (0.3)	10 (0.3)	14 (0.4)	0.392
	**High blood pressure**	1,729 (23.6)	882 (23.9)	847 (23.3)	0.549
	**Cerebrovascular disease**	171 (2.3)	89 (2.4)	82 (2.3)	0.660
	**Immunosuppression**	12 (0.2)	8 (0.2)	4 (0.1)	0.259
	**Chronic lung disease**	309 (4.2)	141 (3.8)	168 (4.6)	0.088
	**Chronic kidney disease**	413 (5.6)	200 (5.4)	213 (5.9)	0.413
	**Heart disease**	574 (7.9)	285 (7.7)	289 (8.0)	0.715
	**Neoplasm**	510 (7.0)	251 (6.8)	259 (7.1)	0.585
**Number of chronic diseases (1–8)**		1.8 (1.04)	1.7 (1.00)	1.08 (1.08)	0.268
**Symptoms**^b^	**General feeling of being unwell (mild or severe)**	3,237 (44.3)	1,816 (49.3)	1,421 (39.2)	<0.001
	**Altered mental state (confusion, stupor, etc.)**	162 (2.2)	96 (2.6)	66 (1.8)	0.023
	**Slurred speech**	214 (3.0)	139 (3.9)	75 (2.1)	<0.001
	**Loss of smell and taste (anosmia, ageusia)**	1,064 (20.0)	701 (27.4)	363 (13.2)	<0.001
	**Fever (≥38°C)**	2,090 (28.6)	1,233 (33.5)	857 (23.6)	<0.001
	**Cough**	2,642 (36.1)	1,389 (37.7)	1,253 (34.5)	0.005
	**Dyspnoea**	1,101 (15.1)	571 (15.5)	530 (14.6)	0.291
	**Pleurisy**	335 (4.6)	162 (4.4)	173 (4.8)	0.445
	**Hemoptysis**	75 (1.0)	40 (1.1)	35 (1.0)	0.609
	**Vomiting**	315 (4.3)	166 (4.5)	149 (4.1)	0.404
	**Diarrhoea**	1,318 (18.0)	700 (19.0)	618 (17.0)	0.030
**Asymptomatic**		2,059 (28.2)	862 (23.4)	1,197 (33.0)	<0.001
**Number of symptoms (1–8)**^a^		2.39 (1.31)	2.43 (1.35)	2,28 (1.26)	<0.001
**Contact with confirmed case**		3,953 (57.5)	2,345 (67.8)	1,608 (47.1)	<0.001

^a^ The full list of symptoms is: fever, cough, dyspnea, general feeling of being unwell, altered mental state, slurred speech, pleurisy, hemoptysis, vomiting, diarrhoea, anosmia / ageusia (loss of smell / loss of taste).

^b^ A case can have one or more Chronic diseases or symptoms at the same time.

### Probability of testing positive for SARS-CoV-2

Table 2 shows the multivariate logistic regression analyses for the variables recorded on each participant’s record sheet. Participants > 45 years of age are more likely to have tested positive for SARS-CoV-2 both when adjusted for gender (OR = 1.17) and when the model is adjusted for the remaining variables (OR = 1.43). Women have a lower probability of having a positive test when adjusted for age (OR = 0.95) and also when adjusted for the rest of the variables (OR = 0.80). When symptoms are considered, the presence of fever raises the probability of a positive SARS-CoV-2 test both when adjusting the model for age and sex (OR = 1.25) and when adjusted for the rest of the variables (OR = 1.49). Participants who presented anosmia and ageusia are also more likely to have a positive SARS-CoV-2 test both when adjusted for age and sex (OR = 1.51) and in the multivariate adjustment (OR = 2.21). A general feeling of being unwell was also associated with a higher likelihood of having a positive SARS-CoV-2 analysis in both models (OR = 1.22 and OR = 1.34, respectively. Finally, having been in contact with a suspected case of COVID-19, even if no symptoms were shown, is also an important predictor of having a positive PCR in the two multivariate models (OR = 1.56 and OR = 2.72, respectively).

**Table 2:** SARS-CoV-2 test results by number of symptoms listed on record sheet^a^.

Variables	Positives (N = 3,686) n (%)	Negatives (N = 3,628) n (%)	OR adjusted to Age and Sex (95% CI)	OR adjusted Multivariable (95% CI) ^b^
**Age**				
**≤ 15**	11 (0.3)	50 (1.4)	0.36 (0.21–0.61)	0.25 (0.09–0.63)
**16**–**45**	1,388 (37.7)	1,596 (44.0)	0.88 (0.84–0.92)	0.47 (0.34–0.64)
**46**–**75**	1,756 (47.6)	1,479 (40.8)	1.15 (1.09–1.20)	(0.53–1.01)
**> 75**	531 (14.4)	503 (13.9)	1.02 (0.96–1.09)	0.88 (0.58–1.34)
**Female**	2,338 (63.4)	2,384 (65.7)	0.95 (0.91–0.99)	0.80 (0.68–0.95)
**Rurality**	888 (24.1)	1,008 (27.8)	0.91 (0.86–0.96)	0.88 (0.74–1.03)
**Socioeconomic level**				
**< 20**	150 (6.4)	216 (9.2)	0.81 (0.72–0.92)	0.78 (0.53–1.16)
**21**–**40**	619 (26.5)	527 (22.4)	1.12 (1.05–1.19)	1.08 (0.81–1.45)
**41**–**60**	1,317 (56.4)	1,361 (57.7)	0.97 (0.92–1.03)	0.92 (0.69–1.19)
**> 60**	187 (8.0)	161 (6.8)	1.09 (0.98–1.20)	1.01 (0.69–1.46)
**Chronical diseases**				
**Diabetes mellitus**	360 (9.8)	315 (8.7)	1.06 (0.99–1.15)	1.23 (0.72–1.53)
**Hepatopathy**	10 (0.3)	14 (0.4)	0.83 (0.52–1.33)	1.21 (0.26–5.59)
**High blood pressure**	882 (23.9)	847 (23.3)	1.02 (0.96–1.07)	1.01 (0.69–1.48)
**Cerebrovascular disease**	89 (2.4)	82 (2.3)	1.03 (0.89–1.19)	1.21 (0.64–2.29)
**Immunosuppression**	8 (0.2)	4 (0.1)	1.32 (0.89–1.98)	0.61 (0.12–3.09)
**Chronic lung disease**	141 (3.8)	168 (4.6)	0.90 (0.79–1.02)	0.78 (0.48–1.27)
**Chronic kidney disease**	200 (5.4)	213 (5.9)	0.96 (0.87–1.06)	0.95 (0.62–1.46)
**Heart disease**	285 (7.7)	289 (8.0)	0.98 (0.90–1.07)	0.71 (0.46–1.07)
**Neoplasm**	251 (6.8)	259 (7.1)	0.97 (0.89–1.07)	0.97 (0.65–1.47)
**Symptoms**				
**General feeling of being unwell (mild or severe)**	1,816 (49.3)	1,421 (39.2)	1.22 (1.17–1.28)	1.34 (1.05–1.71)
**Altered mental state (confusion, stupor, etc.)**	96 (2.6)	66 (1.8)	1.18 (1.04–1.34)	1.25 (0.69–2.26)
**Slurred speech**	139 (3.9)	75 (2.1)	1.30 (1.18–1.44)	1.65 (0.96–2.85)
**Loss of smell and taste (anosmia, ageusia)**	701 (27.4)	363 (13.2)	1.51 (1.43–1.59)	2.23 (1.82–2.74)
**Fever (≥38°C)**	1,233 (33.5)	857 (23.6)	1.26 (1.20–1.32)	1.46 (1.14–1.86)
**Cough**	1,389 (37.7)	1,253 (34.5)	1.07 (1.02–1.12)	1.14 (0.89–1.45)
**Dyspnoea**	571 (15.5)	530 (14.6)	1.03 (0.97–1.10)	0.89 (0.66–1.18)
**Pleurisy**	162 (4.4)	173 (4.8)	0.96 (0.86–1.31)	0.79 (0.52–1.21)
**Hemoptysis**	40 (1.1)	35 (1.0)	1.06 (0.71–1.78)	1.12 (0.46–2.71)
**Vomiting**	166 (4.5)	149 (4.1)	1.05 (0.94–1.17)	0.87 (0.58–1.31)
**Diarrhoea**	700 (19.0)	618 (17.0)	1.07 (1.01–1.13)	0.94 (0.72–1.23)
**Number of symptoms** ^a^				
**0**	1,003 (27.2)	1,246 (34.3)	0.84 (0.79–0.89)	2.63 (0.59–11.61)
**1**–**2**	1,679 (45.6)	1,619 (44.6)	1.02 (0.97–1.07)	1.81 (0.49–6.79)
**3**–**5**	974 (26.4)	741 (20.4)	1.17 (1.12–1.23)	1.76 (0.55–5.62)
**> 5**	30 (0.8)	22 (0.6)	1.15 (0.91–1.45)	0.38 (0.08–1.67)
**Contact**	2,345 (67.8)	1,608 (47.1)	1.56 (1.48–1.64)	2.72 (2.32–3.19)

^a^ The full list of symptoms is: fever, cough, dyspnea, general feeling of being unwell, altered mental state, slurred speech, pleurisy, hemoptysis, vomiting, diarrhoea, anosmia / ageusia (loss of smell / loss of taste).

^b^ The goodness of fit was correct, with similarity between expected and observed values: the Hosmer-Lemeshow test for the multivariate model showed no evidence of a poor fit (p = 0.88).

### Tree diagram to classify cases according to the variables

**Fig 2:**
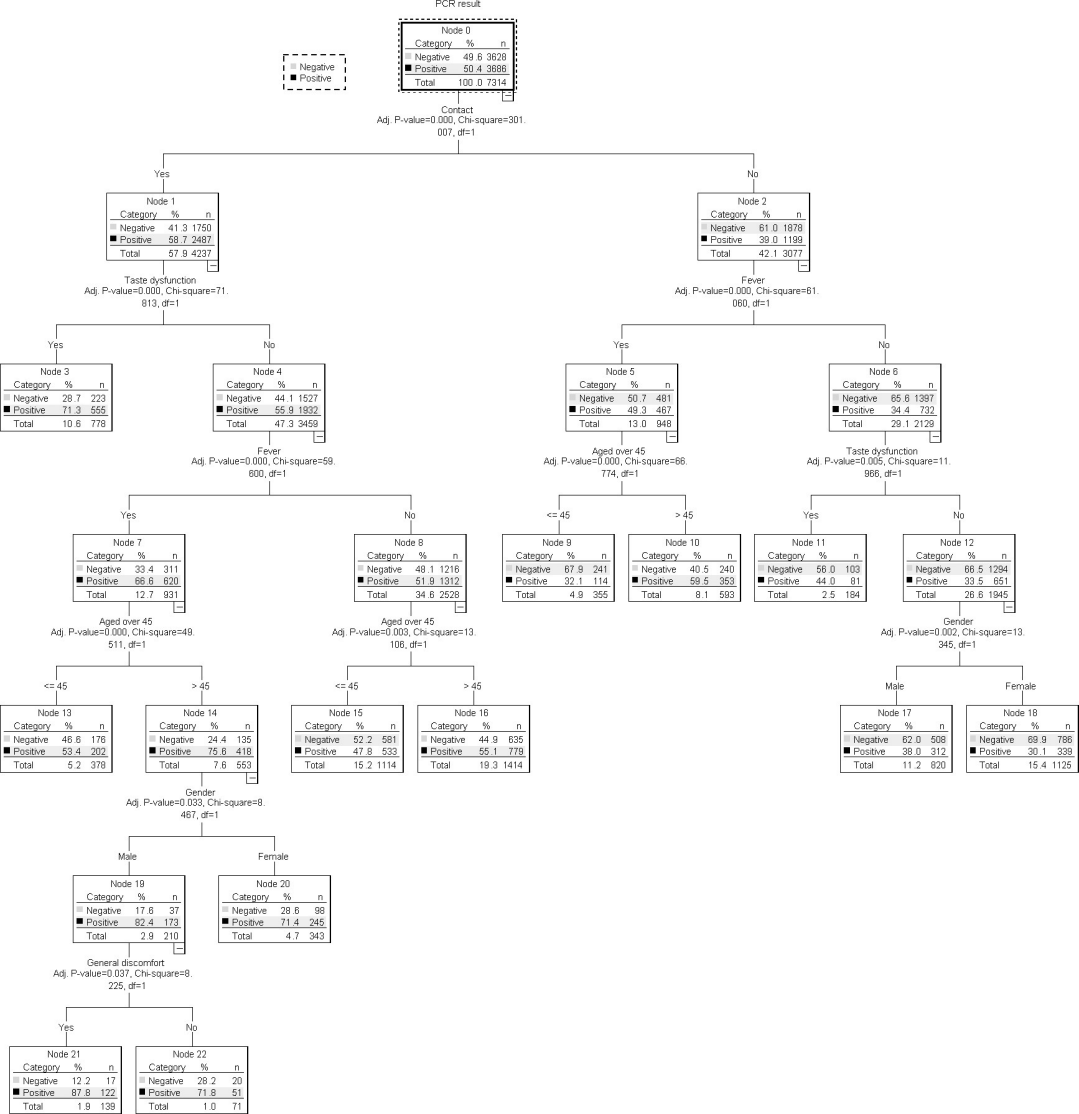
Classification tree diagram of the covariables involved in the proposed model.

The diagram obtained by applying a decision tree is a graphical representation of the model and each possible Boolean alternative corresponding to a decision to be made is represented by a decision node (DN). Each DN contains a table showing the number of cases (frequency and percentage) of the values that the dependent variable under study can take (Fig 2).

The dependent variable (positive PCR) branches into two DN (1 and 2), according to the *Contact* variable, indicating that this is the main predictive variable. The second DN has a higher Chi-square than the first DN (71.81 vs. 61.06) and shows that of the 57.9% cases that are contacts, 58.7% have a positive PCR. The second DN branches back into DN 5 and 6, depending on the presence of taste dysfunction. We observe (DN 5) that cases with taste dysfunction are more likely to have a positive PCR, with a 71.3% compared to 55.9% of DN 6, which is the absence of taste dysfunction. DN 6 branches into DN 11 and 12, depending on the presence of fever. It is observed that a 66.6% of patients with fever have a positive PCR. DN 11 branches into 15 and 16, which correspond to an individual aged over 45. DN 16, which are cases > 45 years of age, has a positive PCR of 75.6%. This DN 16 branches into DN 19 and 20 depending on whether the participant’s gender is male or female. DN 19, which corresponds to males, has a higher percentage of positive PCRs (82.4%). Finally, DN 19 branches between 21 and 22, which defines the presence of a general feeling of being unwell. DN 21, which corresponds to a feeling of being unwell, carries a probability of having a positive PCR higher than the other node (87.7%). The sequence of DNs that best define cases with a positive PCR (variables which influence having a positive PCR) are: DN 0, DN 2, DN 6, DN 11, DN 16, DN 19, DN 21. This is, they influence, in order of importance, the following variables: Contact, Taste dysfunction, Fever, Age > 45, Sex and General feeling of being unwell.

### Multivariable model versus tree diagram

**Fig 3:**
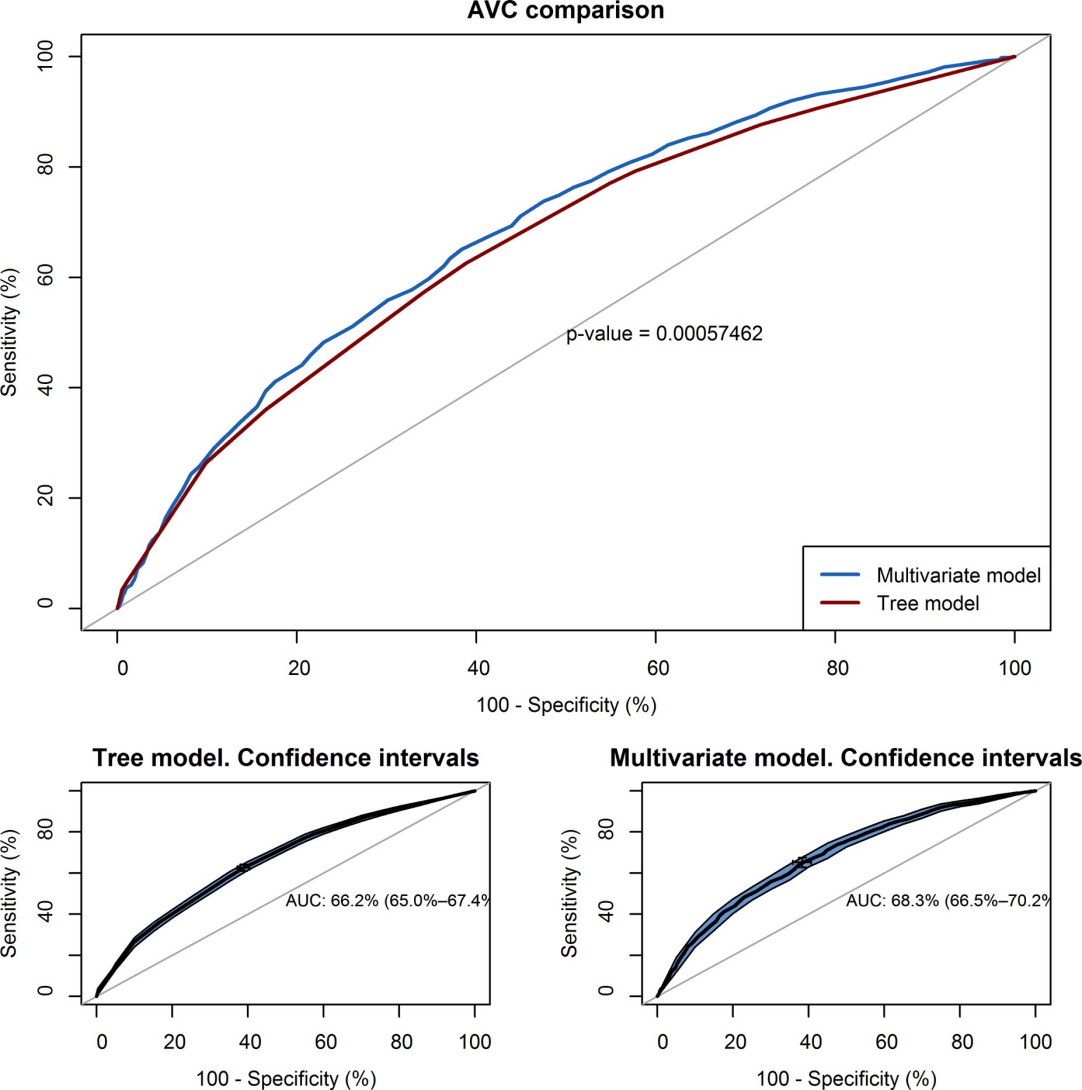
AUC curve showing the relationship between the sensitivity and specificity of the proposed models.

The graphical representation of the area under the curve (AUC) of the multivariable regression model and tree classification (Fig 3), show a similar effectiveness to discriminate between classes. The multivariable model has a slightly higher area under the curve (AUC = 0.68; IC 95%: 0.66–0.70) than the tree model (AUC = 0.66; IC 95%: 0.64–0.68) to predict positive SARS-CoV-2 testing. Through the DeLong test, the “Z” statistic and its associated “p” were calculated, which in this case is Z = 3.47 and p = 0.0005, so we can affirm that the two curves are different. The maximum value of the Youden index obtained with the tree model is 0.27% which corresponds to a sensitivity of 64.3% and a specificity of 62.5%.

## Discussion

This study contributes to improving the detection and classification systems of suspected cases of COVID-19 infection through case-specific demographic and clinical characteristics. The absence of thorough studies [7, 9–11] and the lack of clinical experience regarding this infection [1, 19] generates considerable disagreement between the various authors who propose different models depending on the characteristics and where the study is carried out.

In order to obtain the objective, we have applied logistic regression and decision tree classification methods [20], which show that the variable “Contact with a suspected/confirmed case” is the factor that can best predict the presence of a positive PCR. The highest probability of having a positive PCR (87.8%) occurs among the cases which present concomitantly the antecedent of a contact with a confirmed case of COVID-19, the absence of taste/smell dysfunctions, with fever, aged over 45, male and with the presence of an associated general feeling of being unwell.

The results obtained with the tree diagram technique or logistic regression are very similar. In both methods the variable with the highest gain and proportion of gain is the same, thus with both approaches the root node or main variable is the fact of having documented contact with a confirmed case of COVID-19. Among the variables which refer to the symptoms associated with the presence of COVID-19 infection, both models consider the presence of taste/smell dysfunction, the presence of fever and a general feeling of being unwell as key predictor variables.

Pre-symptomatic and asymptomatic transmission play a key role in the transmission of diseases between communities, especially due to the hidden nature of the spread. If we add to this the fact that the main predictor variable is the contact with a suspected case, the model would guarantee that, apart from a symptomatic diagnosis, the best way to detect a case would be to study contacts. Transmission through contacts and case detections through confirmed case tracking is well documented [21–23]. In addition, the data suggests that some people may be positive for COVID-19 between 1–3 days before developing symptoms, making transmission of the virus more likely in the absence of significant symptoms [24].

### Limitations

This study has several limitations which may affect the interpretation and generalization of the findings. The first is the assumption that the PCR (reference test) presents, in real conditions, a sensitivity and specificity of 100%. Although there is still no consensus, various authors are providing data which could indicate that the sensitivity of the PCR test may not reach 100% in real conditions [25–27]. We must also consider that, although the study period was carried out in the peak phase of the pandemic in Catalonia, the prevalence of the disease was not very high (some seroprevalence studies estimate that this is between 5–7%) [28] and this would make the predictive value of the test very low and therefore some of the positive PCRs in the study could be false positives.

A second limitation is the study population which had a PCR. A large number of individuals whose data were recorded on the screening sheet were excluded from the sample because no confirmatory PCR was performed and this was because during the peak of the pandemic (study period) the PCR performance criteria were limited to a specific sector of the population (vulnerable workers, essential service workers and residents of nursing homes) [28] and this led to the exclusion of most cases during this period. This bias in the sample could cause a model that underestimates the real weight of the predictor variables on the total population and not just a specific sector of it. On the other hand, this type of approach explains the characteristics of the data analyzed in the first wave of the virus, which are different from the current second wave that the country is experiencing.

On the other hand, if we analyze the results of the AUC, in terms of the probability of having a positive test in a suspected patient, we observe that in both models there is a low capacity (66 and 68%) to discriminate between classes and a low sensitivity and specificity.

### Comparison with previous works

The recent emergence of this virus means that many data mining and artificial intelligence projects are currently underway, and although previous studies exist [7, 9, 11, 29], many use hospital data [30], mobile applications for the self-recording symptoms [31, 32] and others are oriented to assessing the risk of admission or complications [8, 33] but few have been performed in the primary care sector with significant data sets [34] to guide the diagnosis of infection and to integrate these solutions into the patient’s medical history [35]. To overcome the limitations described above, it would be necessary to be able to carry out additional analytical-experimental research using more exhaustive methods, extending the range of time and types of cases in the study in order to be able to confirm and expand its results.

## Conclusions

The use of a tree classification model may be useful in screening for the presence of a case of COVID-19 infection. The main variable associated with the risk of developing COVID-19 is the contact with a previously infected person. Early detection and isolation of contacts seems to be the best tool to detect and reduce the extent of SARS-CoV-2.

## Supporting information

S1 File (R)
